# When extubation turns fatal: Delayed hemoptysis from negative pressure pulmonary hemorrhage

**DOI:** 10.1016/j.rmcr.2025.102312

**Published:** 2025-10-17

**Authors:** Mohammed AbuBaha, Hossam Salameh, Wael Hashem, Hasan Khalili, Bara Abubaha, Mohammad Bdair, Hatem M. Taha

**Affiliations:** a Department of Medicine, An-Najah National University, Nablus, Palestine; b Head of Medical Department, Palestine Medical Complex, Ramallah, Palestine; c Teaching Faculty, Faculty of Medicine and Health Sciences, An-Najah National University, Nablus, Palestine

## Abstract

Negative pressure pulmonary hemorrhage (NPPH) is a rare but serious complication following upper airway obstruction, often due to forceful inspiratory efforts.

We report a case of a 25-year-old diabetic male who developed sudden hemoptysis and dyspnea after routine varicocelectomy. The patient had likely bitten the endotracheal tube during extubation, causing transient airway obstruction. Imaging showed bilateral pulmonary infiltrates consistent with alveolar hemorrhage. Other causes were excluded, and supportive treatment led to full recovery.

This case underscores the importance of recognizing NPPH early in the postoperative period to prevent misdiagnosis and ensure prompt, effective management.

## 1 Introduction

Negative pressure pulmonary hemorrhage (NPPH) is a rare but life-threatening complication that usually occurs after an episode of either acute or chronic upper airway obstruction, the proposed mechanism involves the generation of markedly negative intrathoracic pressures which is caused by patient's effort to forcefully inspirate against obstructed airways, the increased negative intrapleural pressures affect the integrity of the alveolo-capillary membrane, which in turn can result in both negative pressure pulmonary edema (NPPE) and in more severe cases, negative pressure pulmonary hemorrhage (NPPH) due to capillary stress failure and increased transcapillary pressure gradients [[1], [2], [3], [4]].

Clinical presentation of patients with NPPH includes hemoptysis, hypoxemia, pink frothy sputum, new pulmonary infiltrates on imaging, and patients often present in the immediate postoperative period, but, as reported, in some cases, it can also have a delayed onset several hours after the inciting event [2,4], While NPPE is more commonly recognized, the occurrence of NNPH is rare but usually present with significant increase in morbidity and mortality risk if not promptly identified and managed [1,3,4].

The literature focused on the importance of rapid recognition and intervention, in addition to being alert in dealing with patients who have risk factors for upper airway obstruction [1,3,4].

Management in most cases is usually supportive, generally focusing primarily on airway protection and oxygenation [4]. In some severe cases, advanced interventions may be used, including mechanical ventilation or extracorporeal membrane oxygenation (ECMO) [4].

Here we describe a case of a 25-year-old male who developed massive hemoptysis and shortness of breath a few hours after an elective low-risk surgery, which turned out to be consistent with signs and symptoms of NPPH, evidenced by the characteristic respiratory signs and symptoms and confirmed with appropriate imaging and exclusion of possible differential diagnoses. The patient was treated conservatively and ultimately discharged on the third day of admission.

## 2 Case presentation

A 25-year-old Middle Eastern male, a known diabetic currently controlled with insulin and glargine, who came for varicelectomy, with unremarkable preoperative evaluations, has undergone an uneventful surgery. A few hours later, during his recovery in the post-op unit, the patient suddenly developed severe shortness of breath (SOB) complicated by hemoptysis described as low in amount. His oxygen saturation readings dropped to 88 % on room air but improved to 95 % with oxygen supplementation, along with a random blood sugar (RBS) measurement of 300 mg/dL.

The surgery went smoothly with successful induction using propofol, fentanyl, and rocuronium. A 7.5 mm endotracheal tube was inserted and ventilation was managed using pressure-control settings. During extubation, neostigmine and glycopyrrolate were given to reverse the neuromuscular blockade. As the patient started to regain spontaneous respirations, he became agitated and bit the endotracheal tube, potentially causing a partial airway obstruction. This may have resulted in forceful inspiratory efforts against the occluded tube possibly leading to pulmonary damage.

**Fig. 1:**
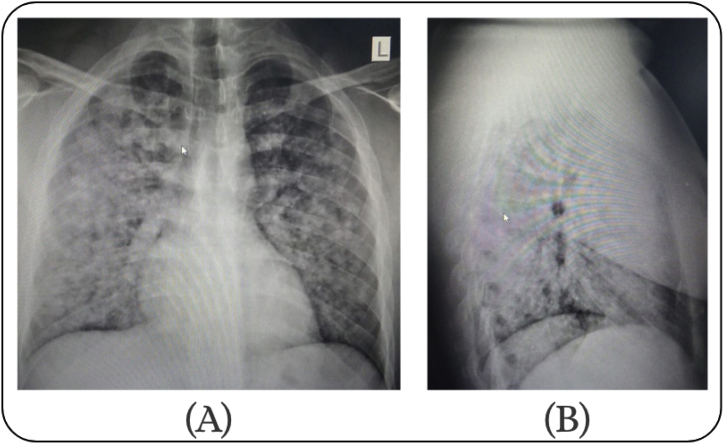
Posteroanterior and lateral Chest X-ray [A,B] images show diffuse bilateral hazy opacities, predominantly in the middle and lower zones, mostly suggestive of a widespread alveolar or interstitial process.

A chest X-ray was immediately performed and showed diffuse bilateral alveolar opacities resembling a ground-glass appearance as shown in Fig. 1. The patient was therefore kept on nasal cannula and was additionally given nebulized adrenaline and referred to another institution for ICU admission. The patient denied any history of fever, vomiting, or loss of consciousness.

Upon arrival, he was weaned off his oxygen support and was still complaining of SOB, in addition to hemoptysis, cough, surgical site pain, and generalized weakness.

On examination, the patient was conscious, alert, oriented, lying in his bed, looking ill, tachypneic, and in pain. Vital signs were stable except for a low oxygen saturation reading and decreased air entry bilaterally on chest auscultation, which had improved with oxygen support. Arterial blood gases revealed low PaO2 at 71 mmHg. A repeat RBS was 340 mg/dL, for which an insulin drip was initiated. He was also administered 20 mg of Furosemide and was prepared for imaging.

**Fig. 2:**
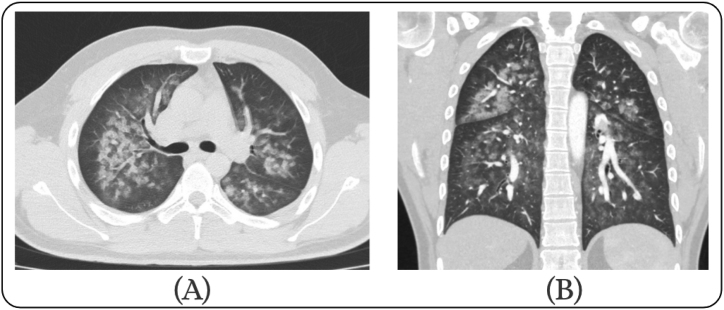
Axial CT [A] and Coronal CT [B] images show diffuse bilateral nodular opacities and interstitial thickening, consistent with diffuse alveolar hemorrhage.

A subsequent high-resolution CT scan was performed and showed bilateral diffuse extensive air space pulmonary opacifications (Fig. 2), which is a highly suggestive feature of pulmonary hemorrhage and less likely to be an infection or edema.

According to these findings, the following differentials were proposed and collected into Table 1.

**Table 1:** List of differential diagnoses.

Differential diagnosis	Description	Rationale
Autoimmune vasculitis	ANCA-associated, Anti-glomerular basement membrane disease (Anti-GBM)	If symptoms progress, further evaluations will be sought
Pulmonary embolism	Can produce similar signs and symptoms and should be considered due to its life-threatening nature	Unlikely due to the negative CT scan findings
Acute respiratory distress syndrome (ARDS)	Can mimic signs, symptoms, and CXR findings, though more aggressive	Unlikely due to the stable hemodynamic status, no precipitating cause, and alert state of the patient
Pneumonia	Aspiration, Post-viral pneumonia	Unlikely due to the clinical picture and consistent laboratory values, and the fact that the patient is covered with antibiotics anyway
Cardiac pulmonary edema	Can produce similar signs and symptoms and should be considered	Unlikely due to the absence of suggestive features like murmurs, crackles, response to diuretics
Bleeding diathesis	Bleeding disorders were ruled out by history and laboratory values	Hemoptysis increasing in frequency or amount indicates the use of nebulized Tranexamic acid (a pro-coagulant) with subsequent bronchoscopy if hemoptysis doesn't improve

The doctors settled on postoperative negative pressure pulmonary hemorrhage (NPPH) as a final diagnosis after ruling out other differentials.

Laboratory data over the first three days of hospitalization revealed a pattern of ongoing blood loss and systemic inflammation. A decline in hemoglobin and hematocrit (16.1–14.8 g/dL and 47 %–43 %, respectively) was noted, reflecting progressive alveolar bleeding. Concurrently, platelet count has dropped (310–232 × 10^9^/L), indicating platelet consumption or bone marrow suppression due to systemic illness which is considered less likely in this setting. A rising white blood cell count (10.6–14.4 × 10^9^/L) and C-reactive protein levels (peaking at 27 mg/L) point toward an acute inflammatory response, most probably due to the pulmonary insult. A complete summary of the laboratory data is provided in Table 2.

**Table 2:** Comparative laboratory data over the course of illness.

Test parameter	Normal range	Results at Day 1	Results at Day 2	Results at Day 3
Complete Blood Count (CBC)				
Hemoglobin	14–18 g/dL	16.1	15.2	14.8
Hematocrit	40 %–54 %	47	44	43
Mean corpuscular volume	80–94 μm^3^	80	81	82
Mean corpuscular hemoglobin	26–32 pg/cell	28	28	28
Mean corpuscular hemoglobin concentration	32 %–36 % Hb/cell	35	35	34
RBC count	4.6–6 million/mm^3^	5.8	5.4	5.3
WBC count	4.6–11 × 10^9^/L	10.6	14.4	11
Platelet count	150-450 × 10^9^/L	310	272	232
General chemistry				
Total bilirubin	0–1.2 mg/dL	0.21	–	–
Creatinine	0.6–1.2 mg/dL	1.1	0.8	1
Blood urea nitrogen	10–50 mg/dL	39	39	–
Sodium	136-145 mEq/L	137	–	–
Potassium	3.5–5.1 mEq/L	4.3	3.9	3.4
Chloride	98–107 mg/dL mEq/L	106	–	–
Arterial Blood Gases				
pH	7.35–7.5	7.37	7.43	7.45
pCO_2_	35–48 mmHg	40.8	45.5	49.4
pO_2_	83–108 mmHg	39	97	82
HCO_3_	22-26 mEq/L	23.6	30.6	34.4
Others				
C-reactive protein	0–6 mg/dL	27	–	18

Based on the overall clinical picture, the following plan of care was implemented: an insulin drip was continued for tighter glucose control, with RBS monitoring every 2 h. Intravenous dexamethasone was initiated to reduce airway inflammation, and furosemide was administered to manage fluid overload and reduce pulmonary congestion. Nebulized ipratropium bromide and budesonide were started to enhance pulmonary function by promoting bronchodilation and decreasing airway inflammation. Oral hydration was encouraged to maintain fluid balance. Anticoagulants were withheld due to concerns about bleeding risk. Ten days later, he returned to the outpatient clinic in stable condition, with no recurrence or residual symptoms.

## 3 Discussion

Pulmonary edema is a well-described clinical entity that is defined as the buildup of transudative fluid, usually due to increased pulmonary capillary hydrostatic pressure as a result of blood backflow from the heart; However, the development of diffuse alveolar hemorrhage or negative-pressure pulmonary hemorrhage (NPPH) as a consequence of disrupted alveolar-capillary stress control is a rarely reported possible complication of increased negative intra-thoracic pressure [5,6].

**Fig. 3:**
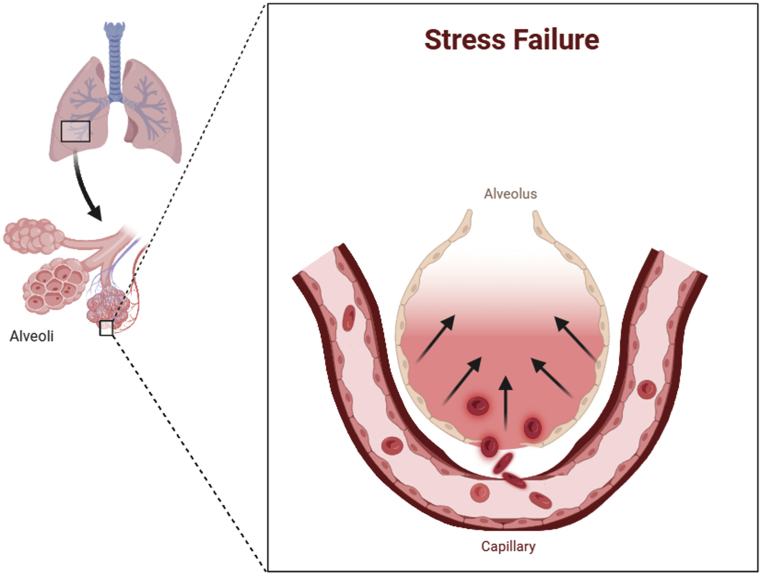
Illustrating stress failure, which is the mechanism by which NPPE develops.

According to Schwartz DR et al., the exact pathophysiology of NPPH is uncertain, though findings can be explained by a disruption to the role of the pulmonary capillaries, or the so-called pulmonary capillary wall stress. West JB et al. describe it as a mechanical failure of the barrier function of the alveolar-capillary membrane, which has been referred to as “stress failure.” [7] as seen in Fig. 3.

NPPH very rarely complicates general anesthesia, with an approximation of 0.05 %–0.01 % of all cases [8]. Many etiologies have been associated with diffuse alveolar hemorrhage (DAH), though it has been well-reported in the literature that the agent (sevoflurane anesthetic gas) was associated with DAH; however, the development of NPPH is usually associated with asphyxiation/strangulation preceding the development of hemoptysis [9].

NPPH diagnosis is usually based on a combination of clinical context, radiologic findings, and bronchoscopic findings [10]. A history of acute upper airway obstruction is considered a key diagnostic feature [11].

This is important because a complication called Negative pressure pulmonary edema (NPPE) occurs when the patient starts inspiring against an acutely obstructed airway, causing a decrease in the thoracic pressure, this intense negative intrathoracic pressure will cause an increase in pulmonary blood flow and an increase in the capillary transmural pressure, resulting in pulmonary edema [11]. NPPE causes restlessness, agitation, cyanosis, and, more characteristically, pink frothy pulmonary secretions [10]. The resulting hypoxemia increases resistance in pulmonary vessels and adversely affects myocardial contractility [12]. All of the latter combined can cause stress failure of the alveolar-capillary membrane, leading to alveolar hemorrhage known as NPPH [12].

**Fig. 4:**
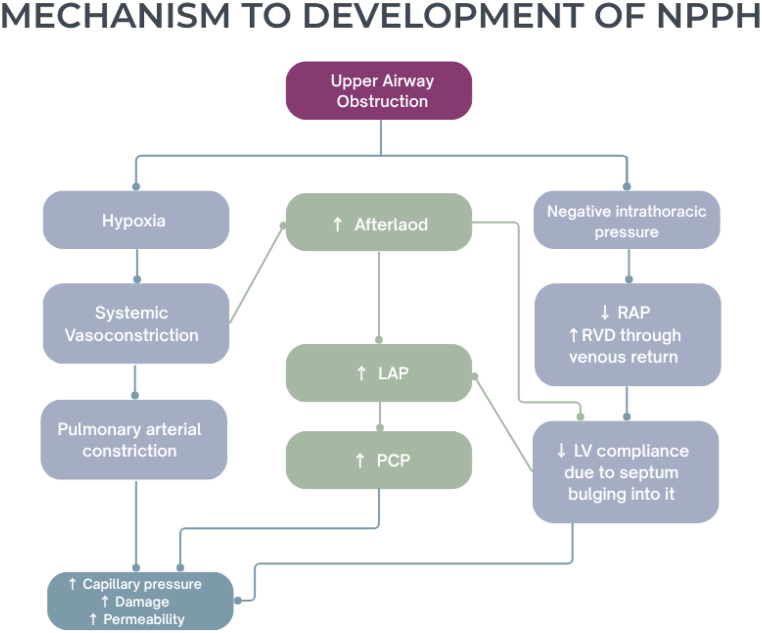
Illustrating the pathogenesis of Negative-Pressure Pulmonary Hemorrhage (NPPH).

Therefore, in order to diagnose NPPH, an integration of the patient's clinical history with clinical findings and imaging is crucial. A more detailed description of the pathophysiology can be seen in Fig. 4 below.

Therefore, etiologies like post-extubation laryngospasm, foreign body, croup, strangulation, epiglottitis, and artificial airway obstruction can all cause NPPE if not appropriately managed with laryngospasm being the most common cause in the post anesthesia care unit [13]. Beside those etiologies, there are multiple risk factors that increase the risk of post-operative airway obstruction and should be addressed if possible, factors like obesity, short neck, airway lesions, obstructive sleep apnea, and acromegaly all increase the risk of NPPE [10].

NPPH is a life-threatening condition that requires immediate attention and management, despite being supportive in nature, management of NPPH hinges on two important priorities: relieving the airway obstruction and vigorous respiratory support [10]. Other measures include hemodynamic stabilization and determining the source and nature of bleeding [6]. NPPH typically shows rapid clinical improvement, often within 24–48 hours, after timely started supporting therapy [10].

Taking actions that prevent airway obstruction reduces the incidence of NPPH, preventive measures like giving lidocaine before extubation help prevent laryngospasm, atropine to reduce airway secretions, and steroids in patients who exhibit risk factors for airway obstruction [10]. it is also important to consider that gentle airway handling, suction of oral secretions, and timely extubation plays a major role in reducing NPPE and NPPH incidence [10].

## 4 Conclusion

NPPH is a rare yet serious under-recognized post-operative event that might complicate any surgery especially in young diabetics. Due to its overlapping features, it can be misdiagnosed as an infection, acute respiratory distress syndrome (ARDS), or even immune-mediated DAH.

Knowledge will definitely help healthcare workers in timely recognition of this devastating complication that can be managed and treated promptly and correctly.

## CRediT authorship contribution statement

**Mohammed AbuBaha:** Conceptualization, Investigation. **Hossam Salameh:** Conceptualization, Validation, Visualization, Writing – original draft, Writing – review & editing. **Wael Hashem:** Conceptualization, Validation, Visualization, Writing – original draft, Writing – review & editing. **Hasan Khalili:** Conceptualization, Validation, Visualization, Writing – original draft, Writing – review & editing. **Bara Abubaha:** Conceptualization, Validation, Writing – original draft. **Mohammad Bdair:** Conceptualization, Supervision, Validation. **Hatem M. Taha:** Project administration, Resources.

## Informed consent

Authors obtained verbal and written informed consent from the patient regarding this case and any accompanying images. A copy of the written consent is available for review by the Editor-in-Chief of this journal on request.

## Disclosures

The authors report there is no competing interests to declare.

## Compliance with ethical standards

All procedures performed in this report involving human participants were in accordance with the ethical standards of the institutional, national research committee, and with the 1964 Helsinki declaration and its later amendments or comparable ethical standards.

## Health and safety

Authors confirm that all mandatory laboratory health and safety procedures have been complied with in the course of conducting any experimental work reported in this paper.

## Funding

The authors received no financial support for the research, authorship, and/or publication of this article.

## Declaration of competing interest

The authors report there is no competing interests to declare.

## References

[1] Negative pressure pulmonary oedema and haemorrhage. https://pubmed.ncbi.nlm.nih.gov/21413434/.

[2] Jo Y., Hwang J., Lee J., Kang H., Hong B. (2021 Mar 16). Negative-pressure-related diffuse alveolar hemorrhage after monitored anesthesia care for vertebroplasty: a case report. J. Med. Case Rep.

[3] Koehler U., Hildebrandt O., Conradt R., Koehler J., Kesper K. (2022 Nov 15). „Negativdruck-Lungenödem“ und „alveoläre Hämorrhagie“ als Komplikationen einer oberen Atemwegsobstruktion. Pneumologie.

[4] Koide M., Kitada T., Kogure M., Fukui K., Sogabe K., Kato Y. (2020 Nov 17;2020). Extraordinary delayed-onset negative pressure pulmonary hemorrhage resulting in cardiac arrest after general anesthesia for vocal cord polypectomy. Case Rep. Critical Care.

[5] Schwartz D.R., Maroo A., Malhotra A., Kesselman H. (1999 Apr 1). Negative pressure pulmonary hemorrhage. CHEST J..

[6] Oswalt C.E. (1977 Oct 24). Pulmonary edema as a complication of acute airway obstruction. JAMA.

[7] West J.B., Tsukimoto K., Mathieu-Costello O., Prediletto R. (1991 Apr 1). Stress failure in pulmonary capillaries. J. Appl. Physiol..

[8] Louis P.J., Fernandes R. (2002 Jan 1). Negative pressure pulmonary edema. Oral Surgery Oral Medicine Oral Pathology Oral Radiology and Endodontology.

[9] Mersh R., Ross C. (2018 Jul 10). Postoperative diffuse alveolar haemorrhage: insidious negative pressure or sevoflurane induced?. BMJ Case Rep..

[10] Han I.S., Han B.M., Jung S.Y., Yoon J.R., Chung E.Y. (2018 Aug 31). Negative pressure pulmonary hemorrhage after laryngospasm during the postoperative period. Acute Critical Care.

[11] Contou D., Voiriot G., Djibré M., Labbé V., Fartoukh M., Parrot A. (2017 Apr 28). Clinical features of patients with diffuse alveolar hemorrhage due to negative-pressure pulmonary edema. Lung.

[12] Kuramoto K., Matsuyama M., Nonaka M., Takeishi T., Oshima H., Matsumura S. (2021 Feb 21). Negative-pressure pulmonary hemorrhaging due to severe obstructive sleep apnea. Int. Med..

[13] Bhaskar B., Fraser J.F. (2011 Jan 1). Negative pressure pulmonary edema revisited. Saudi J. Anaesth..

